# Gluteal liposarcoma presenting as sciatic hernia: A case report and review of literature

**DOI:** 10.1016/j.ijscr.2020.01.015

**Published:** 2020-01-23

**Authors:** Andrew Gomez-Seoane, Tolutope Oyasiji

**Affiliations:** a Department of Surgical Oncology, Barbara Ann Karmanos Cancer Institute at McLaren Flint, Wayne State University, 4100 Beecher Road, Flint, MI 48532, USA; b Department of Surgery, Michigan State University, Lansing, MI, USA

**Keywords:** Liposarcoma, Sciatic, Hernia, Gluteal

## Abstract

**Introduction:**

Lipomatous tumors herniating through the sciatic foramen are extremely rare with less than 6 cases reported in the existing literature. We report a case of gluteal well differentiated liposarcoma that presented as sciatic hernia.

**Presentation of case:**

A 66-year-old female presented with persistent pain in the right gluteal region. Physical examination coupled with CT/MRI scans identified a firm mass extending into the right pelvic side via the sciatic foramen. It measured approximately 18.9 cm × 13 cm × 22.8 cm. The tumor was resected via transgluteal approach. Immunohistochemical and microscopic features were consistent with well-differentiated liposarcoma.

**Discussion:**

Although sciatic hernias associated with intraabdominal contents have been recorded less than 100 times in the literature, instances where gluteal lipomatous tumors manifest as sciatic hernias are even more rare. In this case the gluteal liposarcoma was successfully identified utilizing CT/MRI scanning followed by a transgluteal excision of the mass.

**Conclusion:**

While sciatic hernias are rare, lipomatous/liposarcomatous tumors presenting as sciatic hernias are extremely rare. This case report highlights a combination of rare phenomena. The information presented adds to the current evidence that will guide accurate diagnosis and appropriate treatment of future cases.

## Highlights

• Sciatic hernias are very rare.
• Lipomatous tumors herniating through the sciatic foramen have only been documented in 6 cases.
• CT and MRI imaging of the abdomen and pelvis facilitate diagnosis and appropriate surgical treatment.
• Documented findings add to the current literature and will guide appropriate treatment in future cases.

## 1 Introduction

Sciatic hernias originate from the sciatic foramen and tend to occur less frequently with fewer than 100 cases documented worldwide [1,2]. However, incidence in the past few years has been growing with common symptoms such as intestinal obstruction, intractable pain, or urinary sepsis taking precedence [3]. Clinical diagnosis can be challenging. This is a case of gluteal well-differentiated liposarcoma presenting as a sciatic hernia. This case highlights subtle presentation of sciatic hernia with altered bowel habit in terms of constipation as opposed to classic gastrointestinal symptoms like intestinal obstruction. It also draws attention to the fact that sciatic hernia contents do not necessarily have to be intrabdominal viscera. This unique information contributes significantly to the body of evidence on both liposarcoma and sciatic hernia. Thereby facilitating future prompt diagnosis and treatment of both entities.

This work has been reported in line with the SCARE criteria [4].

## 2 Case report

A 66-year-old female had complained of intermittent discomfort and pain in the right gluteal area. She had also noticed an alteration in bowel habit with recent constipation. The symptoms persisted, and she was treated with analgesia and laxatives.

**Fig. 1:**
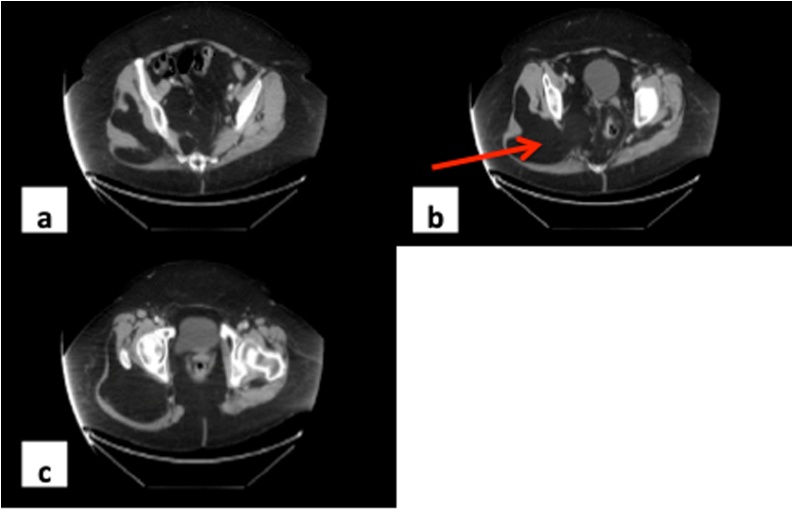
(a) Proximal extent of mass within the right gluteal compartment above the level of herniation into the pelvis. (b) Arrow shows mass herniating through the sciatic foramen into the pelvis and displacing the rectum toward the left (c) Distal extension of the mass within the right gluteal compartment below the level of herniation.

**Fig. 2:**
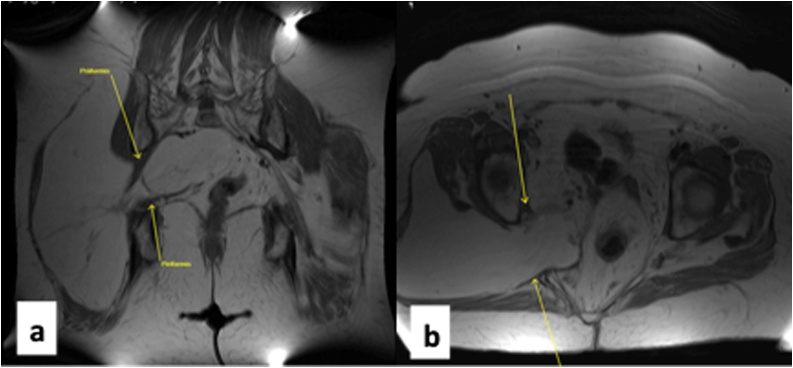
(a) MRI sagittal image of pelvis showing the tumor herniating through the sciatic foramen. (b) MRI axial image showing leftward displacement of the rectum.

**Fig. 3:**
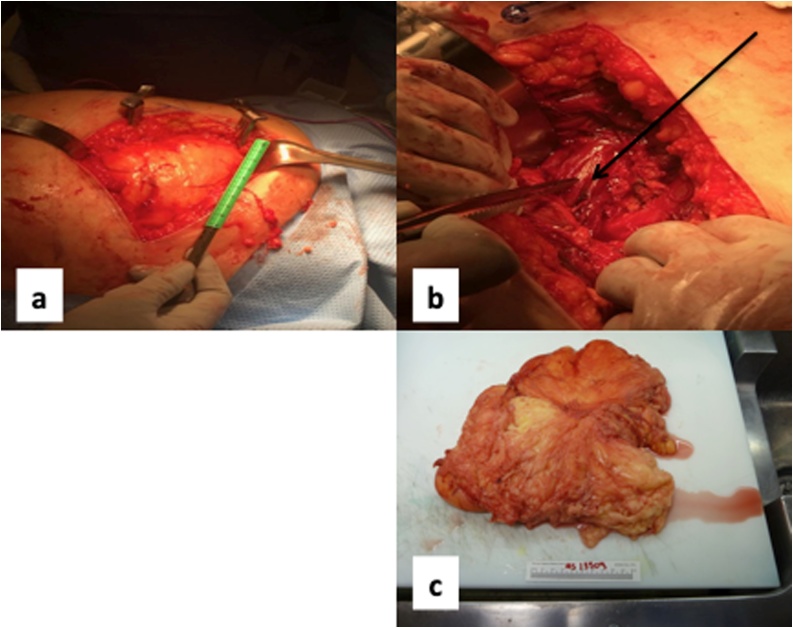
(a) Transgluteal resection, (b) Exposed sciatic nerve after resection, (c) Surgical specimen after resection.

However, there was no specific diagnosis until she fell onto her back and fractured L2, L3 and L4 vertebrae which necessitated further imaging work-up with CT scan of the thoracolumbar spine. Of note, the fractures were stable and non-displaced. Hence, no need for surgical intervention. The CT scan performed to assess the spine revealed an incidental finding of a large mass extending from the right gluteal region into the pelvis. A CT scan of the pelvis was also performed, and this showed a large fatty intramuscular lesion extending into the pelvis through the greater sciatic notch (Fig. 1). The differential diagnosis was listed as a possible lipoma or low-grade liposarcoma. As part of the diagnostic work-up for this right intramuscular gluteal mass with intrapelvic extension, the patient underwent MRI of the abdomen and pelvis. There was no suspicious soft tissue abnormality detected on the MRI of the abdomen but the MRI of the pelvis showed a large homogenous fat lesion noted in the right pelvic side wall between the gluteus maximus and gluteus medius muscles which extended into the pelvis via the greater sciatic foramen (Fig. 2). There was no enhancing component. No heterogeneity or thickened or irregular septations were detected. The margins were distinct. The mass measures approximately 18.9 cm × 13 cm × 22.8 cm. The intrapelvic component of the mass measures approximately 10.5 cm × 6.2 cm × 12.1 cm. There was a leftward displacement of the rectum noticed as a result of the mass effect from the central pelvic mass (Fig. 3).

Physical examination revealed an area of firm induration/possible mass in the lower aspect of the right gluteal compartment. This was not tender and there was no associated redness or discoloration of overlying skin. Digital rectal examination showed normal anal sphincter tone and no mass was palpated within the anorectum.

**Fig. 4:**
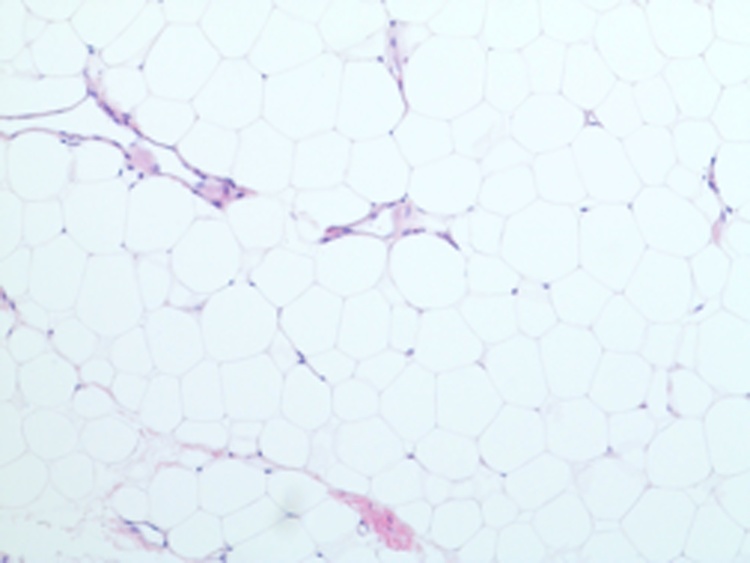
Histology slide demonstrating well-differentiated liposarcoma.

The patient proceeded to have a CT guided core needle biopsy of the mass. This showed scant fragments of fibroadipose and fibroconnective tissue without features of malignancy. There was focal myxoid change. However, the cytologic atypia that would warrant a diagnosis of atypical lipomatous tumor/well differentiated liposarcoma was absent. Furthermore, FISH for MDM2 amplification was attempted, but could not be performed due to sample inadequacy. The case was presented for discussion at the multidisciplinary tumor conference and the consensus recommendation was to proceed with surgical resection of the tumor. Following this, the patient underwent wide excision of the mass through a transgluteal approach and final pathology showed well-differentiated liposarcoma (Fig. 4).

Regarding follow up, the patient has been undergoing surveillance for 3 years. She was seen every 3 months for the first year after resection. Computerized tomographic scans of the chest, abdomen and pelvis were done every 6 months for the first year of surveillance. The patient was seen twice a year for the following 2 years. Computerized tomographic scans of the chest, abdomen and pelvis were done annually for years 2 and 3 of surveillance. The patient has remained disease-free; that is no evidence of local or distant recurrence of the liposarcoma or the sciatic hernia.

## 3 Discussion

Most sciatic hernias have intraabdominal/intrapelvic contents like intestines, greater omentum, ovaries, fallopian tubes, bladder and ureters. According to Skipworth et al., sciatic hernias are extremely rare and have been documented less than 100 times in current medical literature [2]. Sciatic hernias commonly present as gluteal masses associated with compression of the sciatic nerve [1].

In this case the patient did not give any history of classic sciatic pain radiating down the lower extremity. Rather, she complained of localized pain in the right gluteal area. It is speculative to consider that her subsequent fall onto her back (with resultant L2, L3 and L4 vertebral fractures) may be in some way related to sciatic pain in the right lower extremity. While most cases of sciatic hernia will present with gluteal masses, this patient was found to have gluteal mass on imaging which was done for the spine and subsequently confirmed on physical examination. This is most likely due to the fact that the tumor arose within deep intramuscular space between the gluteus maximus and gluteus medius. The patient was also obese, with BMI greater than 35 kg/m^2^. The thickness of the subcutaneous tissue adiposity may have also contributed to decreased prominence of the gluteal mass. Subtle presentation of sciatic hernia with constipation due to compression/ lateral displacement of the rectum attests to the difficulty of diagnosing this condition purely based on clinical signs and symptoms [5]. Of note, following resection of the tumor, the patient’s complaint of constipation resolved. It is also known that when hernia content includes intestines, there can be associated abscess formation in the gluteal region from the strangulation or perforation of involved bowels.

Case reports of lipomatous tumors herniating through the sciatic foramen are extremely rare indeed. To the best of our knowledge the first of such cases was documented by Kerry et al. in 1964 [6]. There were additional 5 cases reported between 2006 and 2015 [2,5,[7], [8], [9]]. This case report will be the seventh case in the published literature. Of all seven cases, five were documented as benign lipomatous tumor; one was documented as atypical lipomatous tumor and this case report is a well-differentiated liposarcoma (Table 1). Descriptive terms like “reverse sciatic hernia” or “inverse sciatic hernia” have been used to highlight the fact that these tumors (lipomatous sciatic hernias) arose within the gluteal compartment and herniated through the sciatic foramen into the pelvic cavity. This is contrary to the more frequently observed phenomenon of herniation from the abdominal or pelvic cavity through the sciatic foramen into gluteal compartment, for non-lipomatous-tumor sciatic hernias [10].

**Table 1:** Summary of previous case reports.

Cases	Author, Year of diagnosis	Age	Gender	Presenting symptoms	Diagnostic imaging	Surgical approach	Pathology
1.	R. Kerry et al., 1964	57	F	Left gluteal mass	Myelogram	Transabdominal and transgluteal	Lipoma
2.	R. Skipworth et al., 2005	36	F	Right gluteal mass	MRI scan	Transabdominal and transgluteal	Atypical lipomatous tumor
3.	E. Guerado et al., 2006	56	Not stated	Left gluteal mass and problem with defecation	MRI scan	Transgluteal approach	Lipoma
4.	E. Lopez-Tomassetti Fernandez et al., 2012	50	F	Constipation, pelvic pain, rectal tenesmus w/o bleeding	CT/MRI scan	Transgluteal	Lipoma
5.	A. Dulskas, 2013	53	M	Right gluteal painful mass, back pain, dull pressure with urgent urination and defecation	CT scan	Transabdominal	lipoma
6.	S. Duran et al., 2015	39	F	Left leg pain and difficulty walking	MRI scan	Transabdominal and transgluteal	Osteolipoma with mature lipocytes
7.	A. Gomez-Seoane, T. Oyasiji, 2019	66	F	Discomfort/pain in right gluteal region, constipation and right gluteal mass	CT/MRI scan	Transgluteal	Well-differentiated liposarcoma

Clinical diagnosis can be quite challenging. Hence, the need for high index of suspicion. Closer attention should be paid to pelvic, gluteal and radicular pain to the lower extremity, even in the absence of obvious gluteal mass. The role of imaging in the diagnosis is cardinal. Currently, CT scan and MRI scan of the abdomen and pelvis are the leading diagnostic imaging modalities [11].

Computerized tomographic scans coupled with MRI are useful in identifying underlying tumors/masses. They also help to identify hernia contents. These modalities are also invaluable in defining surrounding or related anatomic structures. This helps to preoperatively evaluate the surgical techniques for repair and oncologic adequate resection (R0) as necessary. That is, transabdominal, transgluteal or combined approach [11,12]. For this patient, the CT and MRI scans were able to define the hernia content as a lipomatous tumor and generated differential diagnoses of benign lipoma and possible well differentiated liposarcoma. This informed the decision to perform a CT-guided core needle biopsy for tissue diagnosis. A diagnosis of liposarcoma warrants more extensive resection to achieve R0 oncologic resection compared to benign lipoma.

Most lipomas are classified by their proliferation of mature adipocytes with a benign structure in contrast to deep seated lipomas of the pelvic region which tend to grow more slowly as their invasive nature would demonstrate [13]. Some authors believe that neuromuscular diseases along with hip pathology play a role in the development of such sciatic hernias [8]. Distinguishing between well-differentiated and poorly differentiated cell structure is critical in the determination of proper surgical methods for appropriate resection [2]. Present treatment for such tumors has been documented to include a variety of surgical procedures that may utilize transabdominal approach, transgluteal approach or a combination of both. [1,9]. MRI and CT imaging allow for precise diagnosis of the tumor in question and better definition of surrounding anatomical features which facilitate adequate oncologic resection.

## 4 Conclusion

We report a case of gluteal well-differentiated liposarcoma, which presented as sciatic hernia. While sciatic hernias are rare, lipomatous/ liposarcomatous tumors presenting as sciatic hernias are extremely rare. This case report highlights a combination of rare phenomena. So far, there are only six published cases of lipomatous/atypical lipomatous tumors that presented as sciatic hernias. A systematic approach to diagnosis, adequate oncologic resection of the well differentiated liposarcoma as well as treatment of the sciatic hernia via transgluteal approach are documented in this report. This information adds to the current evidence that will guide accurate diagnosis and appropriate treatment of future cases.

## Sources of funding

This research did not receive any specific grant from funding agencies in the public, commercial, or not-for-profit sectors.

## Ethical approval

This study is exempt from ethical approval in our institution.

## Consent

Written informed consent was obtained from the patient for publication of this case report and accompanying images. A copy of the written consent is available for review by the Editor-in-Chief of this journal on request.

## Author contribution

Tolutope Oyasiji MD, MRCSI, MHSA, FACS: Study concept, literature review, drafting of the manuscript, review of the manuscript for important intellectual content and final approval of the version to be submitted.

Andrew Gomez-Seoane BA: Study concept, literature review, drafting of the manuscript and final approval of the version to be submitted.

## Registration of research studies

This study is a case report.

## Guarantor

Tolutope Oyasiji MD, MRCSI, MHSA, FACS.

## Provenance and peer review

Not commissioned, externally peer-reviewed.

## Declaration of Competing Interest

Nothing to disclose.
